# Headspace Injection Method for Intermittent Sampling and Profiling Analyses of Volatile Organic Compounds Using Dielectric Barrier Discharge Ionization (DBDI)

**DOI:** 10.1021/jasms.4c00475

**Published:** 2025-03-11

**Authors:** Daniel Heffernan, Frederik Oleinek, Ayla Schueler, Paak Wai Lau, Jürgen Kudermann, Alina Meindl, Mathias O. Senge, Nicole Strittmatter

**Affiliations:** † Department of Biosciences, TUM School of Natural Sciences, Technical University of Munich (TUM), 85748 Garching, Germany; ‡ Catalysis Research Centre (CRC), Technical University of Munich (TUM), 85748 Garching, Germany; § Department of Design and Green Engineering, Salzburg University of Applied Sciences, 5438 Kuchl, Austria; ∥ Institute for Advanced Study (TUM-IAS), Technical University of Munich (TUM), 85748 Garching, Germany; ⊥ School of Chemistry, Chair of Organic Chemistry, Trinity College Dublin, The University of Dublin, Trinity Biomedical Sciences Institute, Dublin, D02R590 Ireland

## Abstract

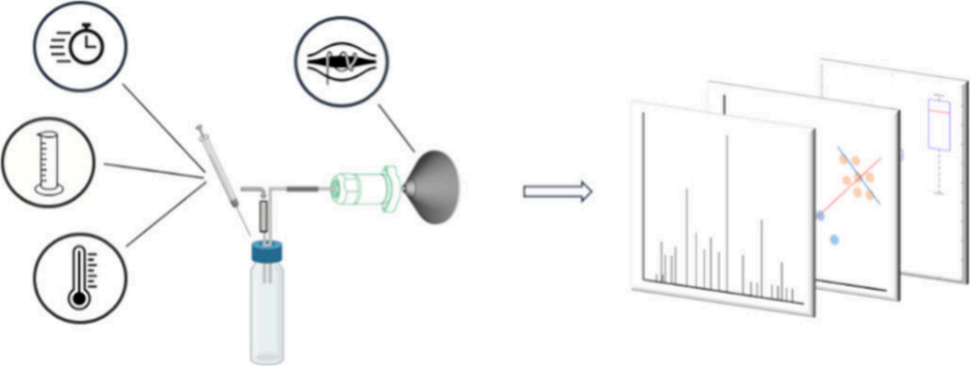

A direct headspace injection method is presented and optimized for the analysis of volatile organic compounds (VOCs) using dielectric barrier discharge ionization-mass spectrometry (DBDI-MS), incorporating an intermediate vial in which the sample headspace is injected. The setup is built of commonly available, cheap consumable parts and easily enables the incorporation of different gases for generating different ionization atmospheres. The method can be fully automated by using standard GC autosamplers, and its rapid analysis time is suitable for high-throughput applications. We show that this method is suitable for both profiling analysis of complex samples such as biofluids and quantitative measurements for real-time reaction monitoring. Our optimized method demonstrated improved reproducibility and sensitivity, with detection limits for compounds tested in the high nanomolar to the low micromolar range, depending on the compound. Key parameters for method optimization were identified such as sample vial volume, headspace-to-liquid ratio, incubation temperature, and equilibration time. These settings were systematically evaluated to maximize the signal intensity and improve repeatability between measurements. Two use cases are demonstrated: (i) quantitative measurement of ethanol production by a metal–organic framework from CO_2_ and (ii) profiling of biofluids following the consumption of asparagus.

## Introduction

Real-time monitoring of chemical reactions is essential for optimizing processes in research and development, manufacturing, and academic studies. Headspace reaction monitoring in particular offers noninvasive insights into chemical reactions while minimizing the risk of instrument contaminations from high abundance compounds such as starting materials.^1,2^ In clinical settings, real-time biochemical reaction monitoring plays a crucial role in diagnostic assays and point-of-care testing, enabling rapid and accurate biomarker detection.^3−5^ Furthermore, these techniques are invaluable in therapeutic drug monitoring, allowing clinicians to tailor treatment regimens based on dynamic insight into drug metabolism.^2^ This capability is especially relevant in the context of personalized medicine, where precise and effective interventions must be developed based on individualized biochemical profiles. Additionally, the real-time monitoring of dietary interventions using headspace analysis can provide critical data on metabolic responses, offering new dimensions to nutrition research and the optimization of dietary regimens for health management.

Dielectric barrier discharge ionization (DBDI) was introduced in 2007 by Na et al.^6^ and demonstrates characteristics similar to atmospheric pressure chemical ionization (APCI). In DBDI, samples are passively drawn into the ionization source due to the pressure differential at the atmospheric pressure interface (API) of the mass spectrometer; ionization occurs through a cold plasma, and it was shown to enable compound analysis in complex matrixes with minimal fragmentation, often producing the [M + H]^+^ quasimolecular ion.^7−9^ An exception to this is saturated alkanes, where distinctive oxidation is observed producing oxidized ions with the formula [M – (2n – 1)H + *m*O]^+^.^10^ The processes that occur in the plasma are complex, and their exact nature is not well understood due to the presence of many different charged species (electrons, free radicals, photons, ions, etc.).^8,9,11^ According to Henry’s law, the amount of dissolved gas in a liquid is directly proportional to its partial pressure above the liquid,^12^ which enables quantification of analytes in solution via the headspace compartment.^13^ The high selectivity of mass spectrometers means the method can be flexibly adapted to different applications and is useful in the determination of unknown sample compositions.^14^ This technique has previously been employed in the analysis of biological materials such as honey,^15^ fruit juice,^16^ and wine,^17^ but is less commonly deployed for biofluids thus far. However, breath analysis is another common application for direct ionization techniques, where volatile biomarkers are detected by breathing directly into the ion source through a heated transfer line.^18,19^

The analysis and monitoring of biomarkers from biofluid offers promising avenues for the early detection, diagnosis, and treatment monitoring of a number of diseases.^3,20,21^ The soft ionization nature of DBDI is ideal for the analysis of complex biofluids, as the presence of the quasimolecular peak and reduced fragmentation facilitates peak annotation. Urine, as a noninvasive biofluid, presents an attractive source for biomarker discovery due to its ease of collection and metabolite richness.^22^ DBDI-MS was previously deployed in a headspace sampling approach to monitor amphetaminic metabolites in urine.^23^

We have previously used DBDI to monitor the production of aroma compounds produced by fungi, by deploying an open vial approach. In this method, the headspace of the fungal culture samples was introduced directly into the DBDI-MS system through the close proximity of the vial opening to the MS inlet. Similar techniques have been utilized in the authentication of food products such as honey^15^ and yak milk.^24^ Alternative methodologies, including interfacing heated solid-phase microextraction (SPME) fibers with DBDI-MS, have been applied for the analysis of pesticides in grape juice.^16^ Additionally, some recent developments in DBDI-MS have focused on real-time reaction monitoring, particularly for industrial applications. For example, Weidner et al. demonstrated the use of DBDI-MS in online monitoring of thermal food processing, where sample aerosols were continuously collected during the thermal processing of wheat rolls.^25^

With this study, we aimed to develop a versatile and easily implemented headspace injection method to mitigate issues related to contamination, reproducibility, and sensitivity of the open vial approach by incorporating the addition of an inert background atmosphere and small-volume sampling. We aimed to demonstrate the applicability of this method to two different application scenarios: (i) intermittent sampling in a model system to produce ethanol (g/L range) and (ii) profiling analysis of complex samples such as urine after a dietary intervention. The optimization process was divided into three critical steps: (1) the sample vial, which contains the sample along with its headspace, (2) the sampling and injection process, and (3) the subsequent MS analysis.

## Materials and Methods

### Chemicals

Ethanol absolute (99.93% purity) and water (LCMS grade) were purchased from VWR. Aroma standards isopentyl acetate, 2-phenylethanol, and ethyl acetate were purchased from Sigma-Aldrich (Merck). dPCN-224(H) metal organic framework was synthesized via the route described by Park et al.^26^

### Sample Preparation

Unless stated otherwise, all experiments were performed with 2 mL of analyte solution in 20 mL headspace vials with a screw cap and silicone-free septum. Sample vials were tested at room temperature with no incubation, being allowed to equilibrate undisturbed for 10 min prior to analysis. Samples that were incubated were placed in an Ohaus (Parsippany, U.S.A.) dry block heater (4 blocks) for 15 min at the designated temperature. Once removed from the heating block, the vials were analyzed immediately.

### DBDI-MS Analysis

DBDI analysis was performed using the SICRIT module by Plasmion (Augsburg, Germany). Unless described otherwise, this module was operated using settings of 1500 V and 15 kHz. The SICRIT ion source was mounted onto an LTQ-XL (Thermo Fisher Scientific, Bremen, Germany) mass spectrometer for ethanol analysis or a Thermo Exactive Classic mass spectrometer for profiling analysis. Capillary temperature was 200 °C. Spectra were acquired in positive ion mode.

Sampling was performed using headspace injection with gastight Hamilton syringes of the 1700 series. Room temperature during analysis was typically between 19 and 22 °C, with humidity ranging from 30 to 40%.

### GC-FID Analysis

GC analysis was performed using an Agilent 7890B gas chromatograph (Agilent Technologies, Santa Clara, California, United States of America) coupled with an Agilent ALS7693/G4513A autosampler and a flame ionization detector (FID). An Agilent VF-200 ms (30 m; 0.25 mm; 0.25 μm) column was used with nitrogen as the carrier gas. Injection volume was 0.6 μL with a temperature gradient of 90, 270, and 320 °C, with an increase at a rate of 15 °C/min.

### DBDI Method Optimization

**Figure 1:**
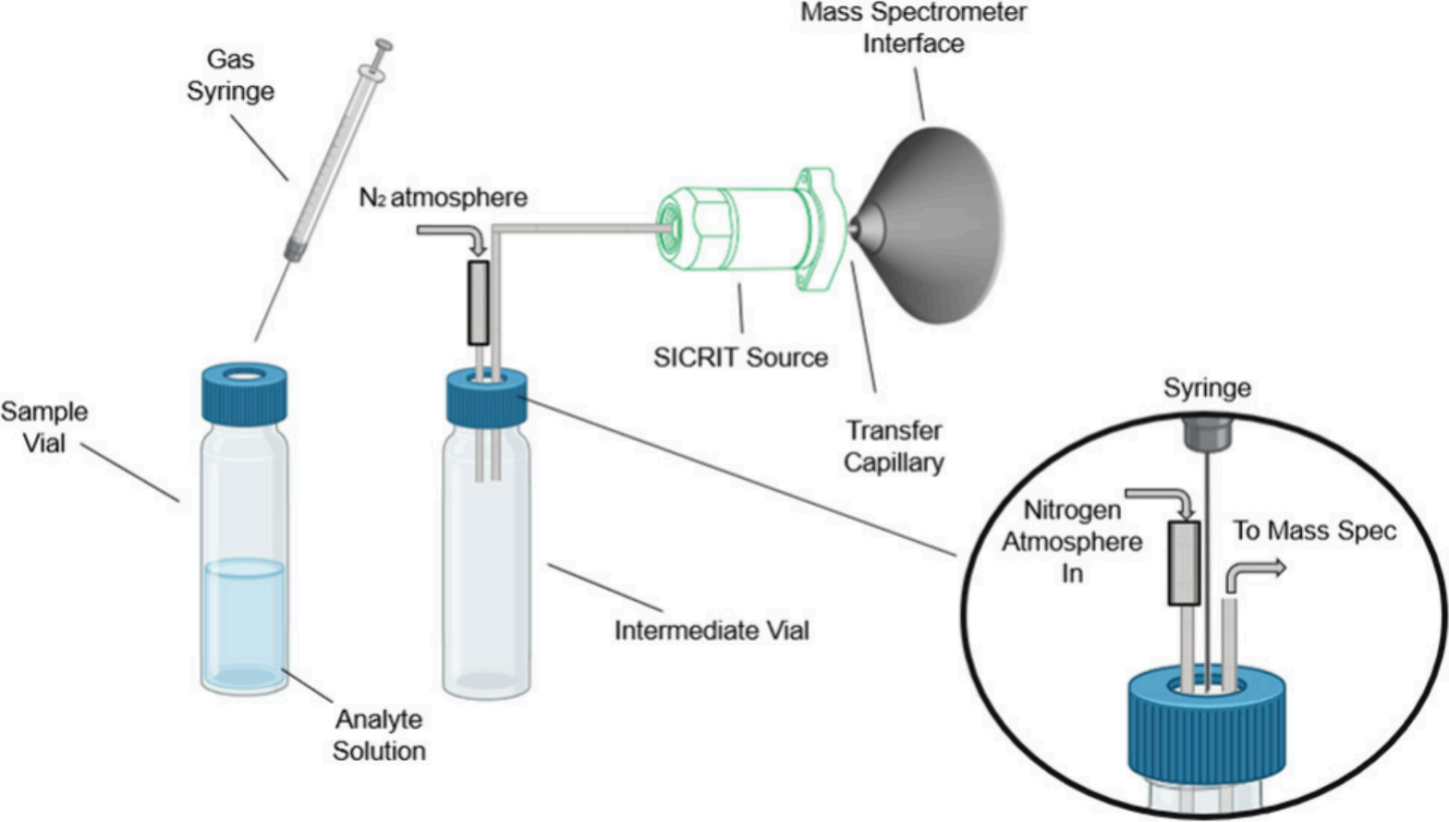
Experimental setup of the headspace injection method. The headspace is aspirated using a syringe and transferred into an intermediate vial for aspiration using the DBDI module. Zoom shows the cap of an intermediate vial with in and outgoing transfer lines.

For method development and optimization, 2 mL of ethanol solution was deposited in 20 mL headspace vials at different concentrations in the mM range to assess the linear range and limit of detection. Figure 1 shows the experimental setup in which the headspace is aspirated from a sealed sample vial and injected into an empty intermediate vial that is connected to the inlet of the DBDI source. A second inlet tube in the same vial enables pressure equilibration.

Due to the small molecular weight of ethanol (46 Da), analysis was performed on a Thermo LTQ XL ion trap mass spectrometer operating at unit resolution in low mass mode (15–200 Da). Maximum injection time was 100 ms, capillary voltage was set to 49 V and the tube lens voltage was 65 V. A mass range of between 25 and 150 Da was used for full-scan spectral acquisition. All analyses were performed in triplicate.

Data analysis was performed using QualBrowser (Thermo Scientific) and Excel (Microsoft). Average absolute intensities for each sample were taken over the entire sampling interval for all analysis discussed. Peak intensity values were plotted against concentrations in Excel.

### MOF Reaction Monitoring

The metal organic framework (placed in the reaction vessel) photocatalytically converts CO_2_ to ethanol in aqueous solution.^27^ A 12 V white LED strip was placed around the reaction vessel to facilitate photocatalysis. Commercially available sparkling water was used as a medium (AVA carbonated natural mineral water). 500 μL sample headspace was injected at a rate of 100 μL/s every 30 min. The total run duration was 3 h. MOF concentration was 16 mg/mL.

### Urine Analysis

Urine was collected in an exploratory study from four participants with an equal male–female split. Food and drinks were available ad libitum and varied between the study participants. Between 50 and 100 g of green asparagus was consumed per person between 5 and 7 pm. Urine was collected at three time points: preconsumption of asparagus (no asparagus consumed for at least 7 days prior to study begin, serving as a baseline control), 3 h postconsumption of asparagus, and 13 h postconsumption (in fasted state following sleep). All specimens were frozen immediately and stored frozen until analysis. Before analysis, samples were aliquoted to 200 uL in 4 mL sample vials (*n* = 4), analyzed in a randomized order with the setup shown in Figure 1, using 1 mL sampling volume. Additionally, 2 pooled samples were generated as quality control specimen. Analysis was performed using four technical replicates. Samples were aspirated and injected at a rate of 250 μL/s into an Exactive classic mass spectrometer operating at a resolution of 50,000 (at *m*/*z* 200), maximum injection time of 200 ms and mass range of 50–300 Da. Samples were tested either at room temperature or subjected to a 15 min incubation at 60 °C.

Principal component analysis (PCA) and analysis of variance (ANOVA) was then performed in Matlab environment, and the top 50 peaks based on *p*-value investigated for their potential to assess changes in metabolite abundance from pre- and postasparagus consumption.

## Results and Discussion

The setup presented here is characterized by its simplicity and low-cost building parts (ca. €150), requiring no specialized parts beyond the DBDI unit itself. It is based on the use of an intermediate vial to inject sampled headspace rather than direct injection into the DBDI ionization module. The main advantage of this setup is the constant pressure at the DBDIionization source, as injection does not lead to inconsistencies in the pressure at aspiration, resulting in a more stable plasma and better reproducibility.

The optimization process of this setup is divided into three parts: (1) the sample vial, which contains the liquid or solid sample along with its headspace, (2) the sampling and injection process, and (3) the subsequent MS analysis. In this section, we present optimization results for each of these and associated settings along the sampling process. Accordingly, the effect of the sample vial will be discussed first, including the sample vial volume, the ratio of headspace to liquid, incubation temperature, and length. Next, parameters affecting the sampling and injection process are discussed, including the injection speed and volume, the volume of the intermediate vial, and the background atmosphere used during analysis. As a third step, instrumental settings affecting MS analysis are discussed. These include the heated transfer line setup, the diameter of the inlet capillary, and the DBDI plasma settings. The optimized method will then be deployed for intermittent sampling and profiling analysis.

We employed a one variable at a time (OVAT) approach for method optimization due to its simplicity and effectiveness in isolating critical parameters. While an alternative approach such as design of experiments (DOE) could possibly provide deeper insights into variable interactions and nonlinear effects, it typically is more difficult to plan and perform correctly and often requires complex data interpretation. Given our primary objective to establish a practical framework for optimizing intermittent sampling and profiling analysis, OVAT allowed for straightforward parameter tuning tailored to the specific requirements of each application.

### Sample Vial

We used ethanol as a model compound for method optimization due to its volatility, low molecular weight, and ubiquity in a laboratory setting. A DBDI mass spectrum of neat ethanol is shown in Figure S1 showing the [M + H]^+^ (*m*/*z* 49.09) and [2M + H]^+^ (*m*/*z* 93) peaks. This [2M+H]^+^ is present only at very high ethanol concentrations and was not observed in dilute solutions. We first investigated the dependence of ethanol signal intensity on the sample vial volume using concentration series and observed a negligible effect on the analyte signal intensity (Figure S2). The concentration of a volatile analyte in the headspace of a vial remains unaffected by increasing the sample vial volumes due to equilibrium dynamics and Henry’s law as long as the sample composition and temperature also remain constant.^28,29^ As the sample vessel volume displayed similar behavior across all volumes tested, a volume of 20 mL was selected due to practical considerations such as sample handling and operational convenience.

Next, we investigated the effect of the headspace-to-liquid ratio in the sample vial using ethanol peak intensities for three different ratios of liquid to headspace (1:10, 1:4, and 1:2). As per Henry’s law, the factors that affect liquid–gas equilibrium are temperature, pressure, and solute concentration.^28,29^ As these three factors remain constant at different headspace-to-liquid ratios, a significant change in peak intensity at each setting was not expected. Indeed, only minor differences in the intensities of each calibration curve are measured (Figure S3). Thus, a ratio of 1:10 liquid to headspace was selected for all further experiments to minimize solvent and reagent usage.

**Figure 2:**
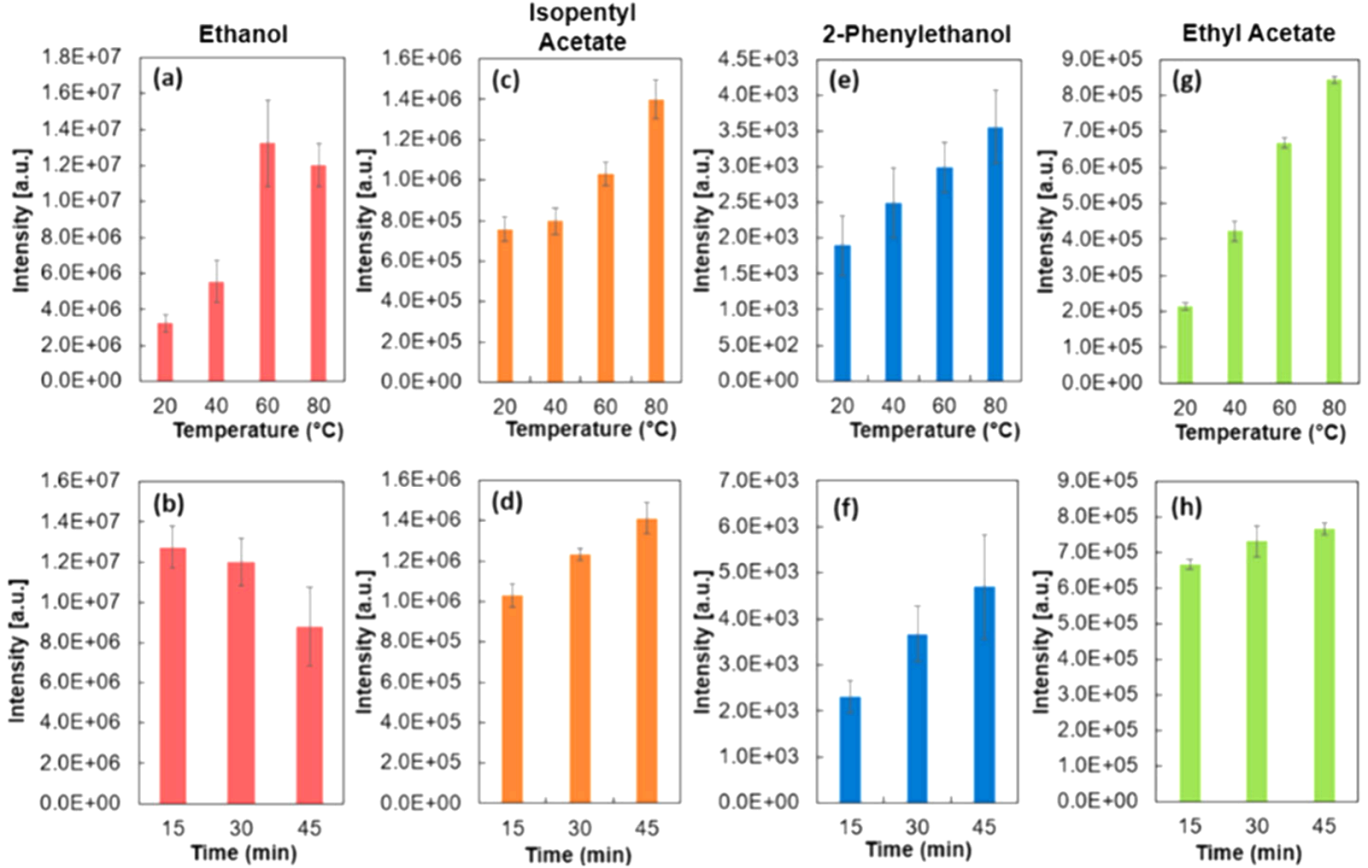
Dependence of signal intensity of ethanol (*m*/*z* 47) with regard to (upper) temperature (25, 40, 60, 80 °C) for 15 min, and (lower) equilibration time (*t* = 15, *t* = 30, *t* = 45) at 60 °C for ethanol (a, b), isopentyl acetate (c, d), 2-phenylethanol (e,f), and ethyl acetate (g, h).

Incubation settings were optimized as a next step, including the incubation temperature and length at four different temperatures and three incubation durations. Here, we expect a strong dependence for compounds with different physicochemical properties, thus we have performed this step for ethanol, and two additional compounds that we have previously studied using DBDI-MS^7^ (ethanol, isopentyl acetate, ethyl acetate). While for ethanol, a minor increase in absolute peak intensity was observed between 20 and 40 °C, an increase of 237% in peak intensity was observed between 40 and 60 °C (Figure 2a). However, a further rise in the temperature to 80 °C resulted in a 10% reduction in signal intensity compared to 60 °C. This decrease is likely attributable to the formation of reaction products arising from the thermal degradation of ethanol, reactions with dissolved oxygen, or interactions between ethanol and water. As shown in Figure S4, the intensity of the *m*/*z* 29 peak exhibits a temperature dependent increase. This peak may correspond to the presence of carbon monoxide (CO) or ethylene (C_2_H_4_), both of which could account for the observed reduction in ethanol peak intensity.^30^

A relatively large standard deviation can be seen for ethanol at 60 °C. As all injections were performed in triplicate and without the use of an autosampler, there exists the possibility of human error with regard to the rate of injection. Indeed, the large error seems to be caused by a single data point if individual data points are displayed (Figure S5). Isopentyl acetate, 2-phenylethanol, and ethyl acetate (Figure 2c, e, and g respectively) exhibited a steady signal increase with elevated temperatures. Performing the incubation at different durations led to the highest ethanol peak intensities at *t* = 15 min reducing by 6% and 36% at 30 and 45 min, respectively. (Figure 2b). The incubation time was found to have less influence than the temperature for isopentyl acetate (Figure 2d) and ethyl acetate (Figure 2h). Isopentyl acetate saw an increase of 38% from 15 to 45 min (14% for ethyl acetate), mirroring optimum settings we found in our previous work using the open vial method.^7^ For ethanol analysis, 60 °C was selected as the optimum incubation temperature. 2-phenylethanol exhibited a steady increase in intensity as incubation time increased, with a 51% increase in signal intensity being observed between *t* = 15 and *t* = 45 min.

To differentiate incubation at elevated temperatures from that performed at room temperature, we use the term equilibration for the latter. Equilibration becomes necessary if the application does not allow for heating of the sample to be tested. Optimizing equilibration time involves balancing the need for providing the sample with enough time to achieve equilibrium with the practical constraints of analysis time and sample throughput. We have examined the effect on ethanol peak intensity and standard deviation for three different equilibration times ranging from 1 to 20 min. At low equilibration times (1 min), large standard deviations are observed for the individual measurements. While the signal is slightly higher at 20 min over 10 min, similar levels of reproducibility are observed (see Figure S6). Thus, we have selected 15 min as an optimal “middle ground” between the considerations of equilibration and throughput.

### Sampling and Injection Process

**Figure 3:**
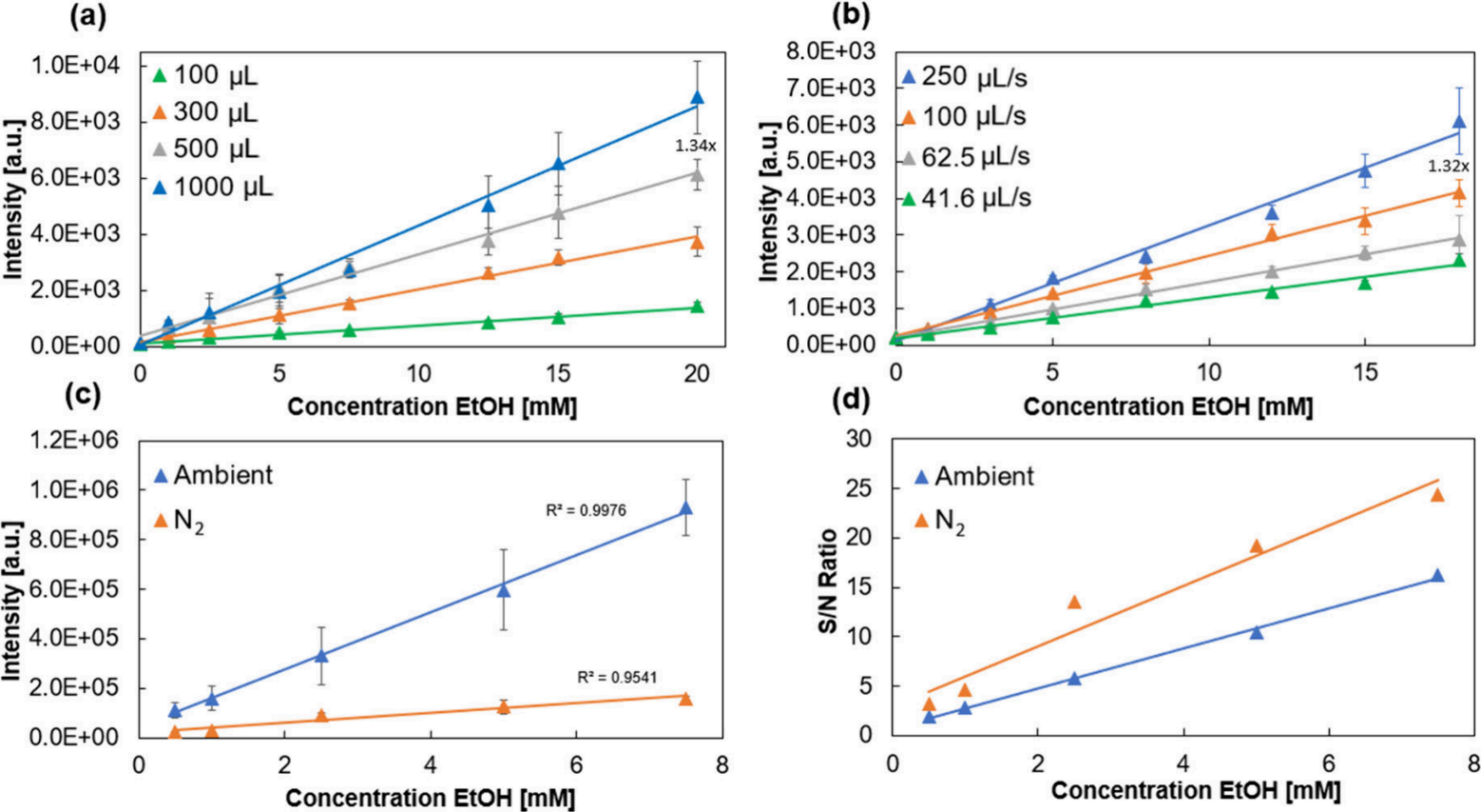
Comparison of peak intensity vs concentration of EtOH for: (a) Increasing injection volume demonstrating a 1.34× increase to signal intensity of 1000 vs 500 μL, (b) increasing rate of injection demonstrating a 1.32× increase to signal intensity of 1000 vs 500 μL, (c) ambient vs nitrogen background atmospheres, and (d) signal-to-noise (S/N) ratio vs concentration of EtOH for ambient and nitrogen atmospheres.

In the next optimization phase, we first studied the effects of the sampled headspace volume and injection speed. As higher sampling volumes result in more material being transferred to the mass spectrometer, we expectedly observed increased signal intensity and thus improved sensitivity at larger headspace volumes (Figure 3a) with intensity increasing 1.34× between 500 and 1000 μL at 20 mM. Thus, if samples were measured only once, 1000 μL was selected as the injection volume.

To investigate the effect of the rate of sample injection, calibration curves of ethanol were analyzed at a constant injection volume of 500 μL, at four different rates of injection, corresponding to total injection times of 2, 5, 8, and 12 s, respectively. As expected, a higher rate of injection led to an increase in the signal intensity (Figure 3b). However, large injection speeds can potentially result in lower reproducibility, especially if injection is performed manually, with the average relative standard deviation (RSD) being 6.9, 8.8, 8.1, and 11% as you move from the slowest to the fastest injection time. However, modifying the injection speed offers opportunities to control the length of the signal obtained. This can be seen in Figure S7, in which the extracted ion chronograms of 250 and 41.6 μL/s are compared, leading to signal durations of 6 and 18 s, respectively. As higher average intensities over the sampling interval are obtained for higher injection speeds, 250 μL/s is chosen for further analysis. The injection volume and speed optimization were repeated for the other model compounds (see Figures S8–S10 for isopentyl acetate, 2-phenylethanol and ethyl acetate). Similar results were obtained to that of ethanol, indicating that the effect on signal intensity is universal and not compound specific.

The effect of the volume of the intermediate vial was assessed by measuring calibration series using five different vial volumes ranging from 2 to 40 mL. Increasing intensities were observed for smaller intermediate vial sizes, with results between lowest and highest intensities showing a 4.25-fold increase at the highest concentration point. This is associated with smaller vials being aspirated faster, resulting in shorter signals of higher intensity due to the reduced analyte dilution in the smaller vials (see Figure S11). We also observed increasing standard deviation as vial size is increased, with the 2 mL vial producing the most consistent results across measurements (0.4–17% RSD), and the 40 mL vial leading to the largest deviations (8–33% RSD). The observed RSD values for the 2–10 mL vial sizes were similar (7–8% RSD) with 4 and 10 mL displaying further very similar intensity levels. However, smaller vial lids create difficulties in installing and positioning the necessary air inlet, MS outlet, and syringe needle into the intermediate vial septum. For this reason, 10 mL N18 cap vials were preferred for this methodology.

The intermediate vial also enabled the implementation of alternative atmospheres for DBDI analysis. It has been shown that reactant ions generated in the DBDI process are highly sensitive to the surrounding atmosphere.^31,32^ Mostly, DBDI is dominated by protonation, particularly under room temperature, dry nitrogen, and humidified nitrogen. However, other reactive ions such as H_3_O^+^, N_2_^+^, and NO^+^ are formed under various conditions and can lead to different ionization mechanisms and increased fragmentation. Weber et al. showed that dry nitrogen led to the highest ionization efficiency and ionization rates through protonation, suggesting that little to no water enhances proton transfer reactions. The sensitivity of a number of different compound classes (aldehydes, ketones, terpenes, alkylphenols, etc.) was also shown to be effected by background atmosphere, with effects as large as 4 orders of magnitude being observed for some of these compounds between the tested makeup gases (room air, dry nitrogen, humidified nitrogen, MeOH, HCl).^32^

We compared DBDI performed at ambient atmosphere to dry nitrogen N5.0 with regard to signal intensity and background analyte levels. Under ambient conditions, a large number of high intensity background peaks are present which may result in interference with the analyte signal during injection due to factors such as ion suppression or masking of the analyte. Oxygen and other reactive gases might further result in the production of oxidized species and a more complex ionization environment. A large reduction in the relative intensity of these background peaks is noticeable under a nitrogen atmosphere. Previous work has shown that a nitrogen rich atmosphere leads to improved ionization efficiency of chlorophenols, alkylphenols, nitrophenols, and alkanes.^32^ In our study, the N_2_ atmosphere is found to have a negative effect on absolute ethanol signal intensity (ca. 80% reduction, see Figure 3c); however, this is offset by a reduction in background levels resulting in a 1.5–2.3× increased signal-to-noise ratio between ambient and nitrogen atmosphere sample series (Figure 3d).

### Analysis Settings

**Figure 4:**
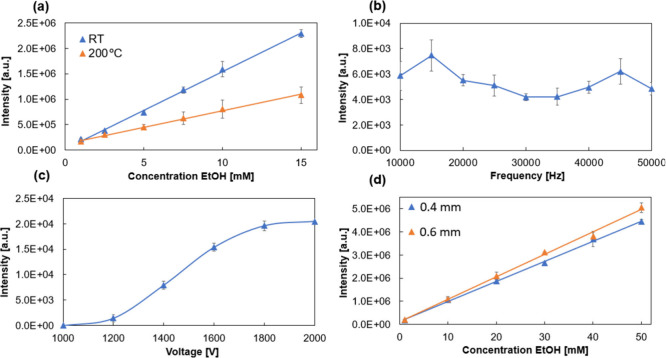
(a) Intensity vs concentration of EtOH with heated and room temperature transfer capillary. Effects of plasma settings on peak intensity, (b) intensity vs frequency, and (c) intensity vs voltage. We have performed our optimization in between the ranges recommended by the manufacturer, voltage of between 1000 and 2000 V, and a frequency of 10–50 kHz. (d) Intensity vs concentration of EtOH with differing MS transfer capillary diameters (0.4 mm and 0.6 mm ID).

Following the path of the analyte to the MS, the next step is transfer from the intermediate vial to the MS. We tested the effect of a heated transfer line using the respective Plasmion Heated Transfer Line, comparing its performance at room temperature and 200 °C (Figure 4a). The experimental results show a 50% reduction in signal intensity upon heating at the 15 mM ethanol point. Furthermore, generally higher standard deviations can be seen at every concentration at this elevated temperature (3–13 vs 11–22% RSD). This result appears to agree with the thermal instability of ethanol that was noticed earlier (Figure 2). Thus, for ethanol analysis, we chose to proceed using plain metal transfer tubing without heating.

Next, ionization in the DBDI source occurs. The plasma processes can be influenced via the frequency and voltage settings in the manufacturer’s control module. The plasma frequency (the characteristic frequency of electrostatic oscillations in the plasma) can play a significant role in determining the efficiency of ionization of the analyte.^33^ The effect of both plasma settings on the ethanol intensity is shown in Figure 4b and c. Ethanol intensity increases with increasing plasma voltage up to the maximum 2000 V, although a saturation effect is visible above 1800 V. An increase in frequency initially leads to an increase in ethanol intensity at 15 kHz. At higher frequencies the intensities show a successive decline up to a value of 35 kHz (reduction of 44% from 15 kHz) at which a second increase is observed with a maximum at 45 kHz. The effect of plasma frequency and voltage are however compound specific. Michael et al. showed that the RF frequency was shown to have a minimal effect on signal intensity within the range of 10–20 kHz, however the plasma voltage was shown to have a significant impact on the ionization efficiency of a number of tested lipid classes, with the optimal voltage being 1500–1600 V.^34^ In the case of perfluorinated compounds, loss of fluoride through reactive oxygen species formed in the plasma could be reduced at higher frequency levels.^35^ Furthermore, the signal-to-noise ratio was shown to be directly influenced by plasma volume and generator power.^36,37^ While ca. 15 kHz displays the highest signal intensity, the smallest standard deviation is observed at 30 kHz which would therefore be more suitable for quantitative analysis. Thus, optimum conditions are application dependent and will require a compromise between sensitivity and reproducibility.

The inlet transfer capillary follows the DBDI module. Two different internal diameters (0.4 and 0.6 mm) were tested for their effect on signal intensity. The internal diameter of the transfer capillary impacts the internal airflow dynamics and, thus, analyte transport into the DBDI source, which is driven by the inherent MS vacuum system. Thus, narrower capillaries result in faster airflow but potentially less analyte at the plasma zone, while wider capillaries offer less restricted airflow and thus an increase in the absolute amount of analyte molecules reaching the plasma. The large diameter capillary was found to have a 2.5× higher signal intensity (at 20 mM ethanol concentration, Figure 4d) due to the increased airflow through the DBDI source. However, an increase in standard deviation is noted with higher analyte concentration in the large diameter capillary (1–7% vs 1–12%). For this reason, the narrow diameter capillary (0.4 mm) was deemed better. The optimized method resulted in a LOD of ethanol from 0.1 mM and a linear range from 1 to 250 mM (see Figure S12).

To compare the performance of the optimized method to that of our previously published open vial method,^7^ we have further determined LOD and linear range for compounds isopentyl acetate (Figure S13), 2-phenylethanol (Figure S14), and ethyl acetate (Figure S15). The linear ranges observed were 0.00125–0.1 mM, 0.005–1 mM, and 0.00025–0.01 mM for isopentyl acetate, 2-phenylethanol and ethyl acetate, respectively, via the direct infusion method. The limits of detection were 0.00025 0.005, and 0.00025 mM in the same order. The lower limit of detection for 2-phenylethanol was disimproved relative to the previously developed method. The moderate volatility of 2-phenylethanol may be the cause of this, as the addition of the intermediate vial may result in the adsorption of the analyte to the sampling syringe, vial surface, or transfer capillary. For our previously developed open vial method, the linear ranges of isopentyl acetate, 2-phenylethanol, and ethyl acetate were found to be 0.00273–0.101 mM, 0.00273–0.0303 mM, and 0.00273–0.0303 mM, respectively. This constitutes an improvement to the width of the linear range of 1.5× for isopentyl acetate, a 20-fold increase for 2-phenylethanol, and 1.24× for ethyl acetate. (see Table S1). The reproducibility of the direct infusion method was compared to our previously developed open vial method.^7^ The open vial method was performed on a series of 15 samples of a solution containing 10 mM ethanol in water and resulted in an RSD of 21.4%. Repeat injections of 500 μL of the same sample concentration (10 mM ethanol) were performed with the direct infusion method at a rate of 62.5 μL/s over 15 injections and resulted in a RSD of 5.6%.

### Applications

**Figure 5:**
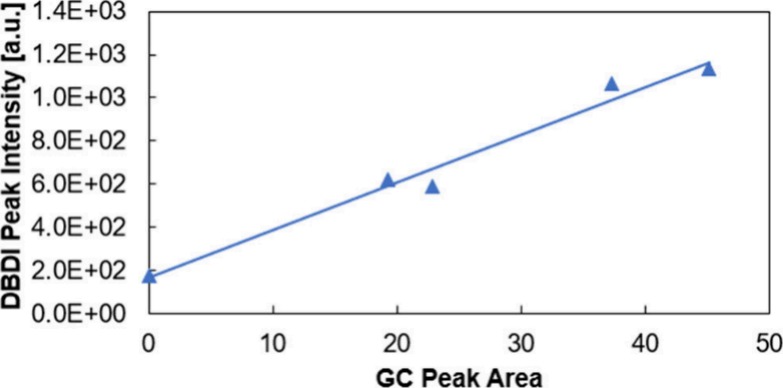
Correlation plot of DBDI ethanol peak intensity vs GC-FID ethanol peak area.

We have performed our optimization studies on ethanol as one of our aims was to develop a method to perform intermittent sampling for ethanol determination in a MOF catalyzed reaction that produces ethanol from CO_2_.^27^ While previous method optimization steps were performed in water, we observed that peak intensities obtained for ethanol in water are roughly 20× greater than in the presence of the MOF (Figure S16) due to ion suppression. However, acquisition of ethanol in the mM concentration range of interest remains feasible. While initially, longitudinal sampling was intended repeatedly from the same reaction vial, we noted that closed systems with small reaction volumes experience depletion of starting materials and products with each sampling event (Figure S17a), resulting ultimately in a decrease in detected converted ethanol after 80 min (Figure S17b). Thus, individual vials were tested to produce a time course in which concentrations continued to increase over time. Good agreement was found between DBDI measurements and the determination of the ethanol concentration from the solution phase using GC-FID (see Figure 5).

To test whether the method can serve as a profiling method for diagnostic or biomarker detection purposes, we next evaluated the suitability of the optimized method (Figure 1) for the profiling of a complex biological sample such as a biofluid. Urine as a widely and noninvasively available biofluid was chosen and analyzed before and after asparagus (*Asparagus officinalis*) consumption in a small scale, exploratory study. Asparagus has long been associated with the generation of characteristic odorous compounds in urine, attributed to sulfur-containing metabolites such as methanethiol and dimethyl sulfide.^38,39^

**Figure 6:**
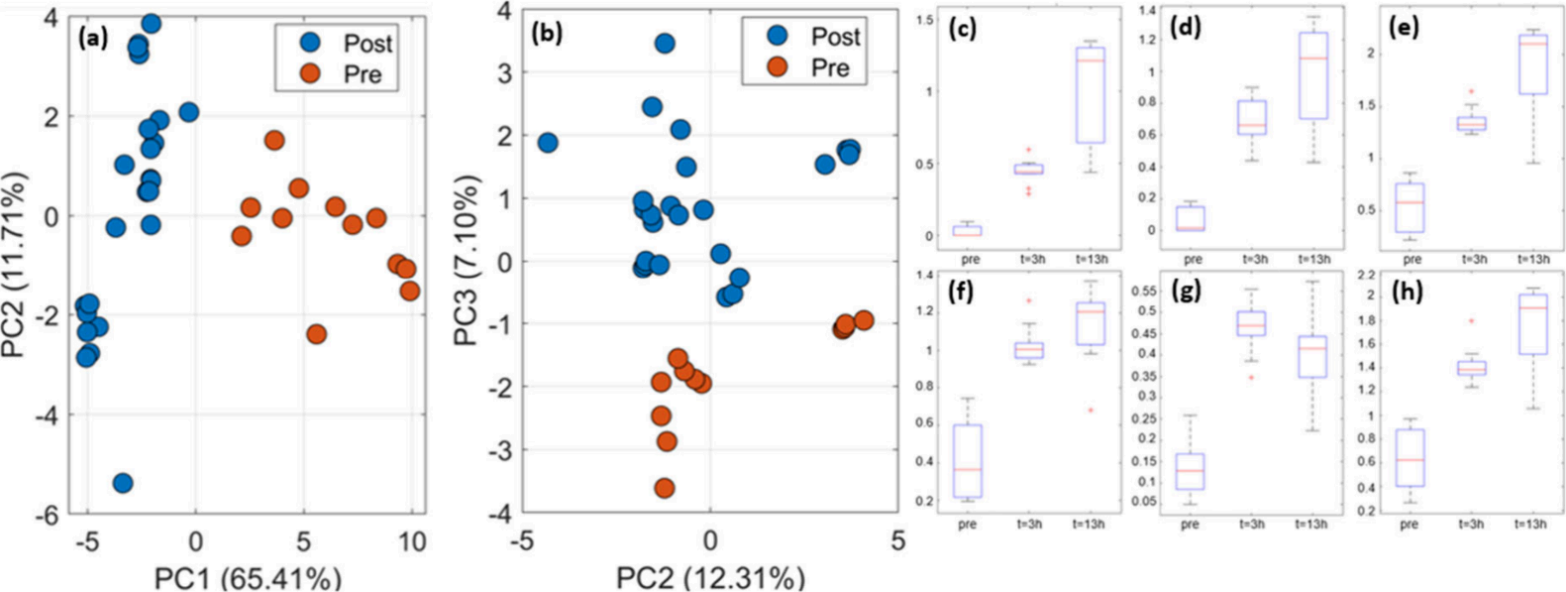
Comparison of PCAs between (a) room temperature and (b) incubated sample sets. Boxplots for pre-asparagus consumption, 3 h post, and 13 h post for *m*/*z* (c) 68.9997, (d) 71.0153, (e) 75.0286, (f) 77.041, (g) 80.0599, and (h) 131.0844.

Urine was analyzed directly without the requirement for dilution or other sample preparation steps beyond headspace equilibration. However, a smaller sample vial and smaller sample volume were chosen (4 mL and 200 μL, respectively) to make the method more suitable for the small sample volumes frequently associated with biofluids. We first revisited the incubation procedure, as in our previous work we observed improved sensitivity of compounds of interest after incubation. We compared the spectra for incubation at room temperature and under increased temperature and observed several peaks that are abundant in the room temperature samples to be of decreased intensity post heating, among these are peaks likely corresponding to urea ([M + H]^+^ - *m*/*z* 61.0671) and urea monohydrate ([M + H]^+^ - *m*/*z* 79.0781) (see Figure S18). Urea in aqueous solution has previously been observed to thermally degrade at temperatures as low as 20–40 °C.^40^ However, other signals such as *m*/*z* 103.1151 and *m*/*z* 121.0649 (2-methyl-6-vinylpyrazine [M + H]^+^) are noted to increase. However, as the goal was to differentiate between samples before and after asparagus consumption, we compared the respective data sets under both conditions using Principal Component Analysis (PCA, Figure 6a,b). A clear separation between pre- and post asparagus consumption can be seen in both plots. However, at room temperature, this separation is observed along PC1, while for incubation at higher temperature, the same separation is not observed along PC1, but instead is only being observed along PC3. This suggests that the incubation step introduces variability into the spectral profiles that are masking the biological effect of interest; potentially, due to the degradation of thermally labile compounds or increased transfer of contaminant VOCs or such VOCs that are unrelated to asparagus consumption at elevated temperature.

ANOVA was then performed on the data, and the top 50 peaks based on *p*-value were inspected more closely. Boxplots generated of these peaks (Figure 6c–h) highlight six peaks that are raised in the post vs the pre consumption samples, with the remaining 44 peaks being diminished post consumption. These were *m*/*z* 68.9997, 71.0153, 75.0286, 77.041, 80.0599, and 131.0844. The peaks at *m*/*z* 77.041, and 75.0286 likely correspond to the [M + H]^+^ adduct of 1-propanethiol and 2-propene-1-thiol, sulfur-containing compounds that are likely a result of asparagus consumption. The peak at *m*/*z* 131.0844 tentatively corresponds to the [M + H]^+^ adduct of 3-methylcyclohexanethiol. All these *m*/*z* values remain elevated after 13 h, when all study participants were in a fasted state following sleep.

## Conclusions

We developed a direct headspace injection method using DBDI-MS incorporating an intermediate injection vial for monitoring chemical reactions via intermittent sampling and profiling of complex samples. We identified those settings that affect analyte intensities such as the rate of injection, ambient atmosphere composition, intermediate vial volume, transfer capillary temperature, plasma frequency, and plasma voltage. These should be optimized on the basis of each compound or each new application, thus giving a blueprint for future method optimization. However, application-dependent restrictions should be noted. The method has good reproducibility, demonstrating a clear improvement on the previously developed open vial strategy with an RSD of 5.6% vs 21.4%. While in the case of profiling analyses, incubation can be favorable, this step might not be applicable to the scenario of reaction monitoring, where the reaction conditions should remain such that optimum reaction rates and product yields are maintained. Our results studying ethanol production in a MOF catalyzed reaction from CO_2_ achieved rapid ethanol quantification with results that closely align with GC-FID measurements from the same samples.

The presented method is further suitable for urine profiling without the need for sample preparation, as demonstrated in an exploratory study following a dietary intervention with asparagus. The methodology is easily automated using an autosampler and injector, similarly to modern GC systems for high throughput testing of clinical samples. Assessing the volatile spectrum from a urine sample has the potential to help clinicians follow nutrition or identify biomarkers of disease in its earliest stages and when treatment interventions are most effective. The potential to collect and compare samples between time points enables the possibility of tailoring therapeutic regimens to individual patients based on their metabolic profile, allowing for personalized care focused on early detection, targeted therapy, and improved patient outcomes.

## Supporting Information Available

Sample spectrum of neat ethanol; dependence of signal intensity to sample vial volume; dependence of peak intensity to liquid-headspace ratio; intensity vs temperature for *m*/*z* 29; dependence of average signal intensity of ethanol (*m*/*z* 47) with regard to temperature for 15 min showing overlay of individual replicates; effect of sample equilibration time on peak intensity and standard deviation; effect of rate of injection on peak width; effect of sample volume and rate of injection on peak intensity for isopentyl acetate, 2-phenylethanol, and ethyl acetate respectively; dependence of signal intensity with respect to intermediate vial volume; linear range and limit of detection for ethanol, isopentyl acetate, 2-phenylethanol, and ethyl acetate respectively; table containing the linear ranges for the aroma compounds measured for the open vial and direct infusion methods; effect of ion suppression on signal intensity; effect of repeat sampling on a single sample on ethanol solution and MOF reaction solution; and PCA and loadings comparison of incubated and room temperature asparagus urine samples (PDF)
