# Supplementary material for: Headspace Injection Method for Intermittent Sampling and Profiling Analyses of Volatile Organic Compounds Using Dielectric Barrier Discharge Ionization (DBDI)
Source: J Am Soc Mass Spectrom. 2025 Mar 11;36(4):801–10. doi: 10.1021/jasms.4c00475 (PMC11969650; doi:10.1021/jasms.4c00475)
Supplement: Supplementary file 1 — js4c00475_si_001.pdf [file js4c00475_si_001.pdf]

SUPPLEMENTARY INFORMATION

Headspace injection method for intermittent sampling and profiling analyses of volatile organic compounds using Dielectric Barrier Discharge Ionization (DBDI)

Daniel Heffernan[1], Frederik Oleinek[1], Ayla Schueler[1], Paak Wai Lau[1], Jürgen Kudermann[2], Alina Meindl[3], Mathias O. Senge[4,5], Nicole Strittmatter[1,*]

1 Department of Biosciences, TUM School of Natural Sciences, Technical University of Munich (TUM), 85748 Garching, Germany

2 Catalysis Research Centre (CRC), Technical University of Munich (TUM), 85748 Garching, Germany

3 Department of Design and Green Engineering, Salzburg University of Applied Sciences, 5438 Kuchl, Austria

4 Institute for Advanced Study (TUM-IAS), Technical University of Munich, 85748 Garching, Germany

5 School of Chemistry, Chair of Organic Chemistry, Trinity College Dublin, The University of Dublin, Trinity Biomedical Sciences Institute, Dublin, D02 R590 Ireland

*Corresponding author: nicole.strittmatter@tum.de

Content*:* Sample spectrum of neat ethanol; dependence of signal intensity to sample vial volume; dependence of peak intensity to liquid-headspace ratio; intensity vs temperature for *m*/*z* 29; dependence of average signal intensity of ethanol (*m*/*z* 47) with regard to temperature for 15 minutes showing overlay of individual replicates; effect of sample equilibration time on peak intensity and standard deviation; effect of rate of injection on peak width; effect of sample volume and rate of injection on peak intensity for isopentyl acetate, 2-phenylethanol, and ethyl acetate respectively; dependence of signal intensity with respect to intermediate vial volume; linear range and limit of detection for ethanol, isopentyl acetate, 2-phenylethanol, and ethyl acetate respectively; table containing the linear ranges for the aroma compounds measured for the open vial and direct infusion methods; effect of ion suppression on signal intensity; effect of repeat sampling on a single sample on ethanol solution and MOF reaction solution; and PCA and loadings comparison of incubated and room temperature asparagus urine samples.

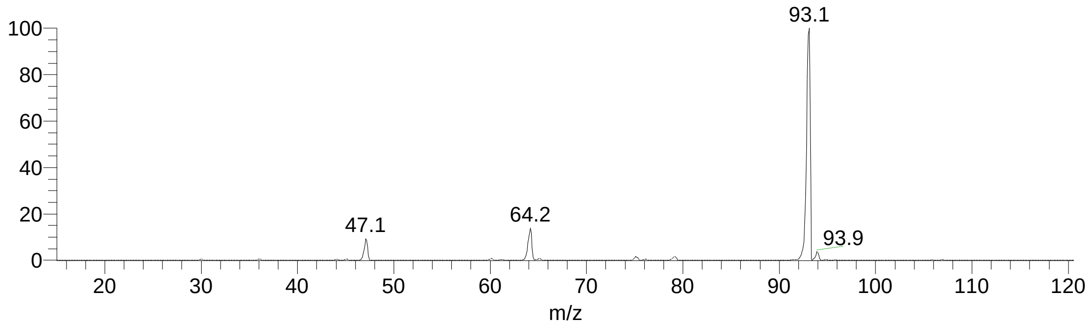

Figure S1. Mass spectrum of pure ethanol recorded with DBDI ion source. The $[M+H]^+$ (*m/z* 47.1) and $[2M+H]^+$ (*m/z* 93.1) are visible**.** The now dominant dimer ion disappears in diluted solutions.

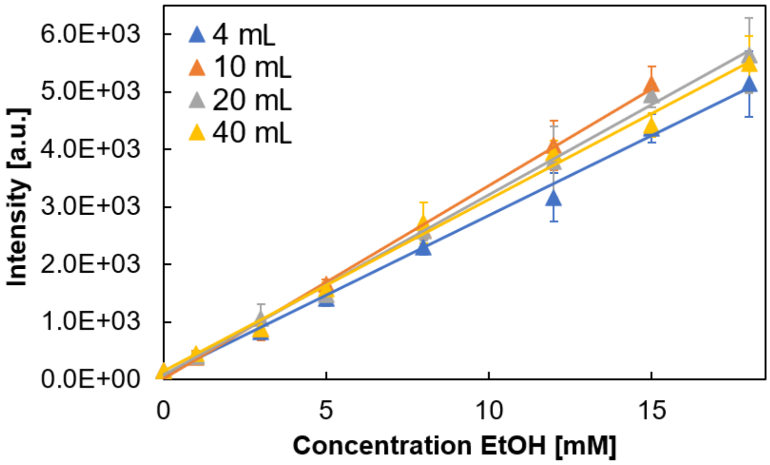

Figure S2. Dependence of signal intensity with regard to sample vial volume. Analyte solution was maintained at 10% of total sample vial volume.

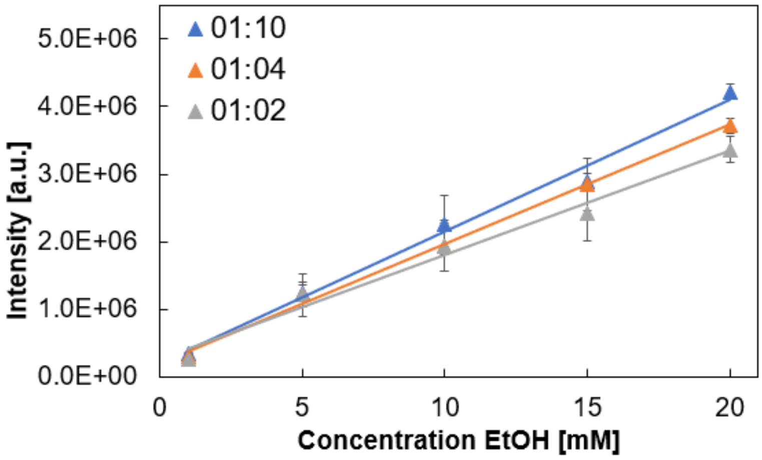

Figure S3. Comparison of peak intensity vs concentration for EtOH for increasing liquid to headspace ratios.

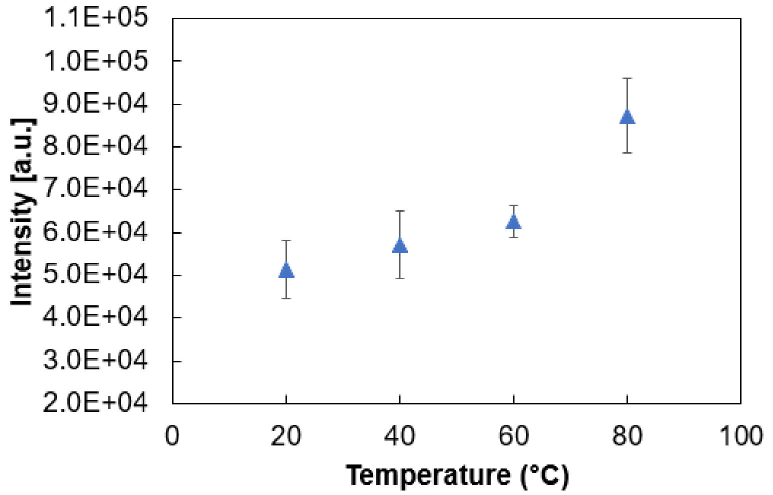

Figure S4. Intensity (a.u.) vs Temperature (ºC) for *m/z* 29 for a 2 mL solution of 10 mM ethanol in water in a 20 mL GC vial.

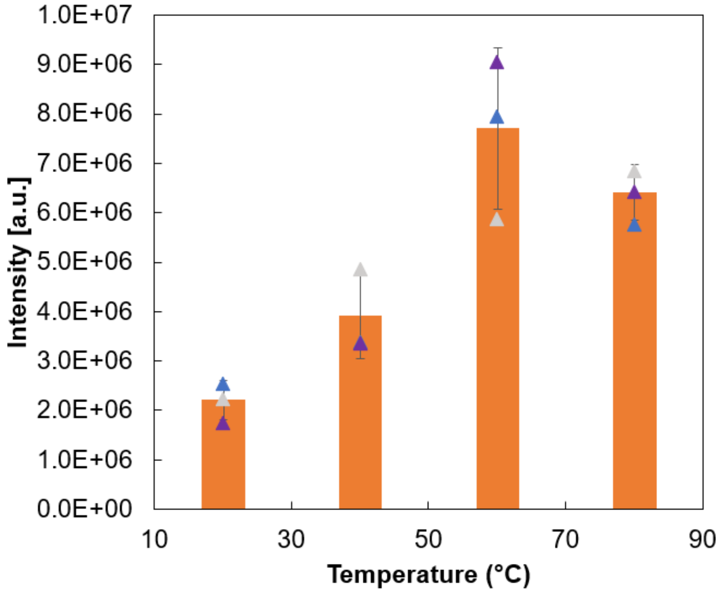

FigureS5. Dependence of average signal intensity of ethanol (*m/z* 47) with regard to temperature for 15 minutes showing position of individual replicates.

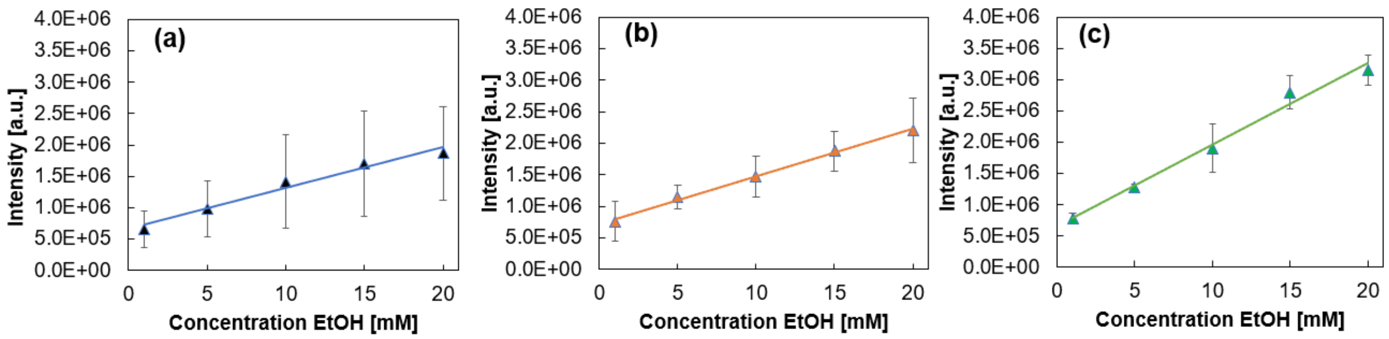

Figure S6. Effect of sample equilibration time on ethanol peak (*m/z* 47) intensity and standard deviation performed with identical calibration curves of ethanol in water at 3 different equilibration times; (a) 1 min, (b) 10 min, and (c) 20 min.

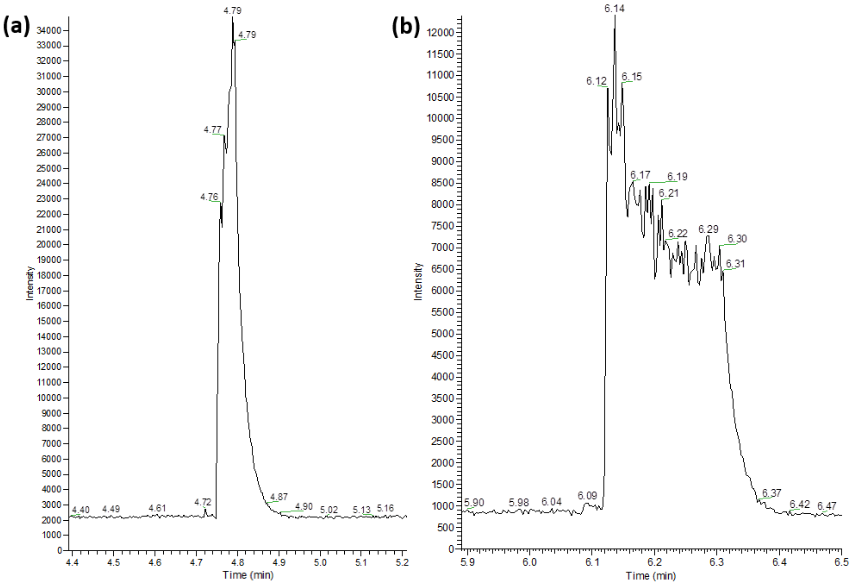

Figure S7. Comparison of peak width between two rates of headspace injection; (a) 250 µL /s (b) 41.66 µL /s.

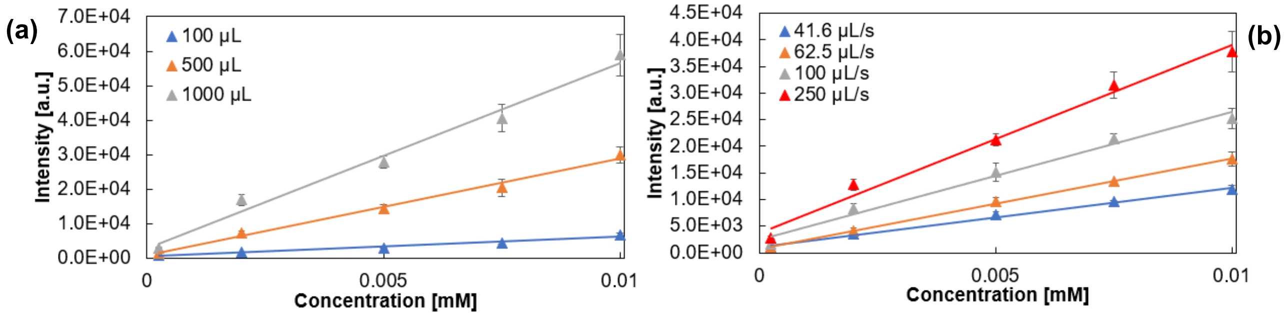

Figure S8. Comparison of peak intensity vs concentration of Isopentyl Acetate for: (a) Different injection volume, (b) different rate of injection.

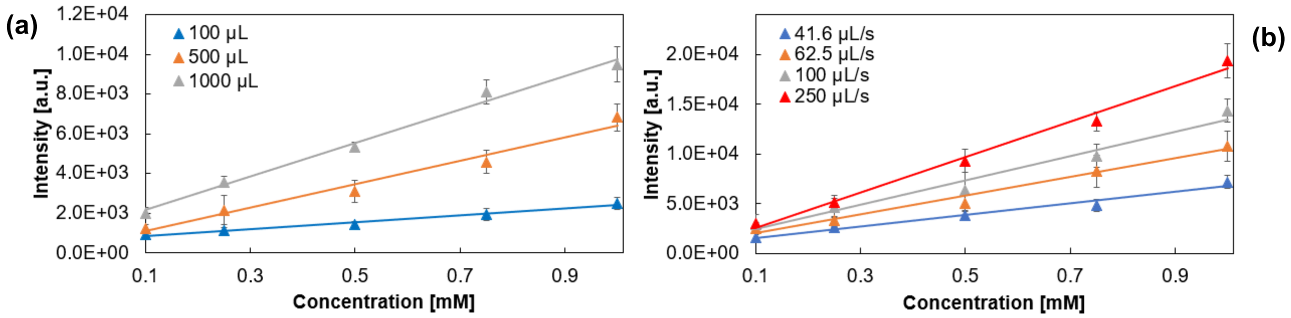

Figure S9. Comparison of peak intensity vs concentration of 2-Phenylethanol for: (a) Different injection volume, (b) different rate of injection.

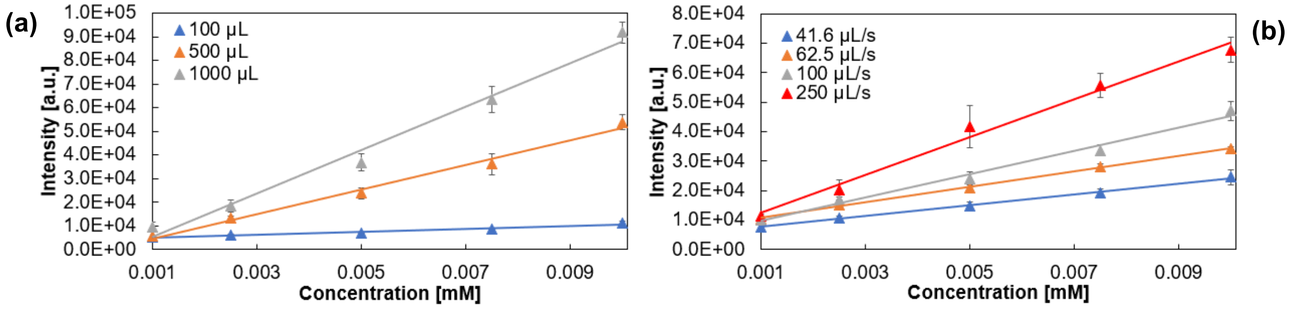

Figure S10. Comparison of peak intensity vs concentration of Ethyl Acetate for: (a) Different injection volume, (b) different rate of injection.

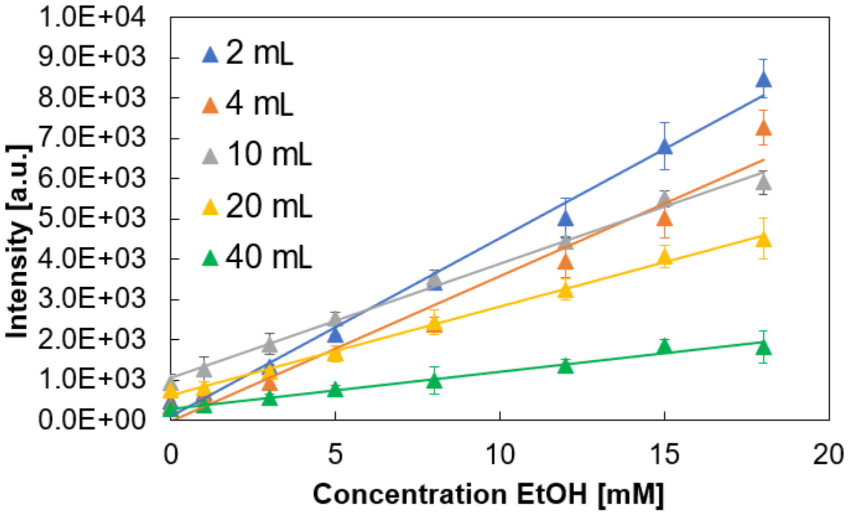

Figure S11. Effect of intermediate vial volume on peak intensity of ethanol performed for 5 intermediate vial volumes on calibration curve of ethanol in water (2 mL sample volume, 20 mL sample vessel volume).

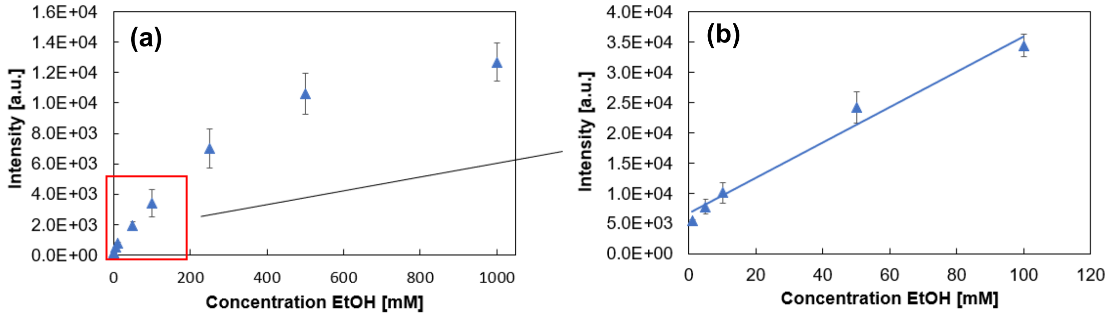

Figure S12. Application of ideal method to identify the linear range and limit of detection for ethanol: (a) whole recorded concentration range, (b) zoom into low concentration ranges marked with red box.

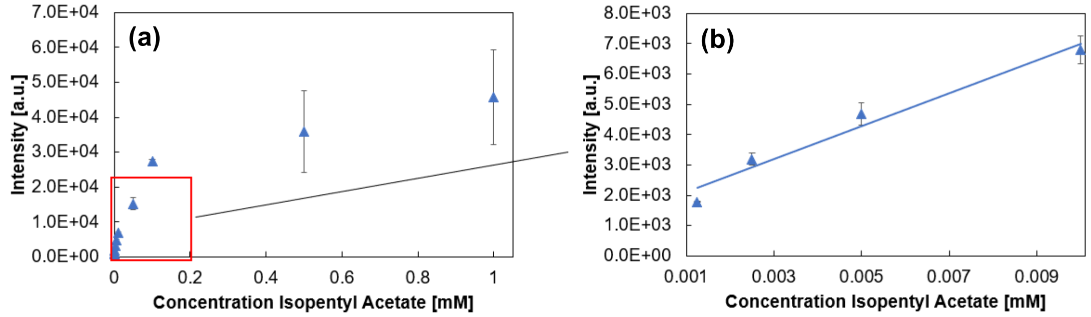

Figure S13. Application of ideal method to identify the linear range and limit of detection for isopentyl acetate: (a) whole recorded concentration range, (b) zoom into low concentration ranges marked with red box.

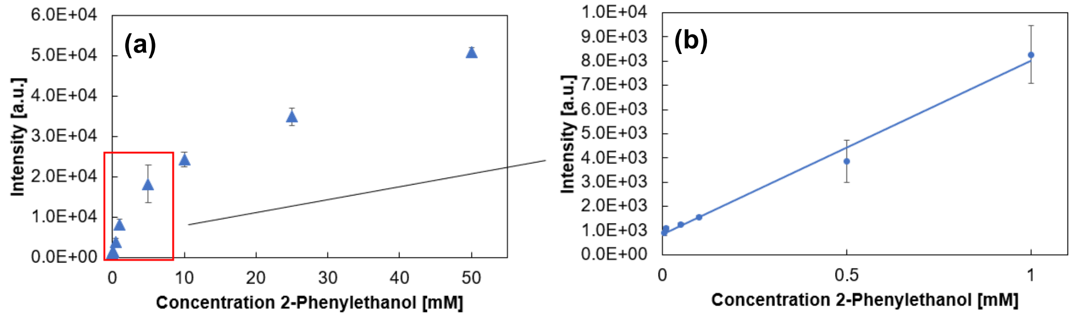

Figure S14. Application of ideal method to identify the linear range and limit of detection for 2-phenylethanol: (a) whole recorded concentration range, (b) zoom into low concentration ranges marked with red box.

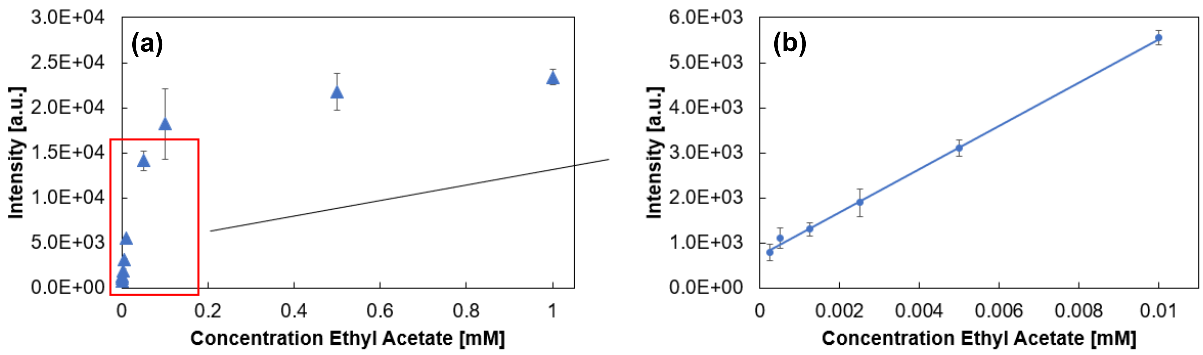

Figure S15. Application of ideal method to identify the linear range and limit of detection for ethyl acetate: (a) whole recorded concentration range, (b) zoom into low concentration ranges marked with red box.

Table S1. List start and end points of linear range of aroma standards tested with method 1 (open vial method) and method 2 (direct infusion method).

Number	Name	Sum Formula	Method 1 Linear Range (mM)	Method 1 Limit of detection (mM)	Method 2 Linear Range (mM)	Method 2 Limit of detection (mM)
1	Isopentyl Acetate	C7H14O2	0.00273 - 0.1	0.00273	0.00125 - 0.1	0.00025
2	2-Phenylethanol	C8H10O	0.00273 - 0.03	0.00273	0.005 - 1	0.005
3	Ethyl Acetate	C4H6O2	0.00273 - 0.03	0.00273	0.00025 - 0.01	0.00025

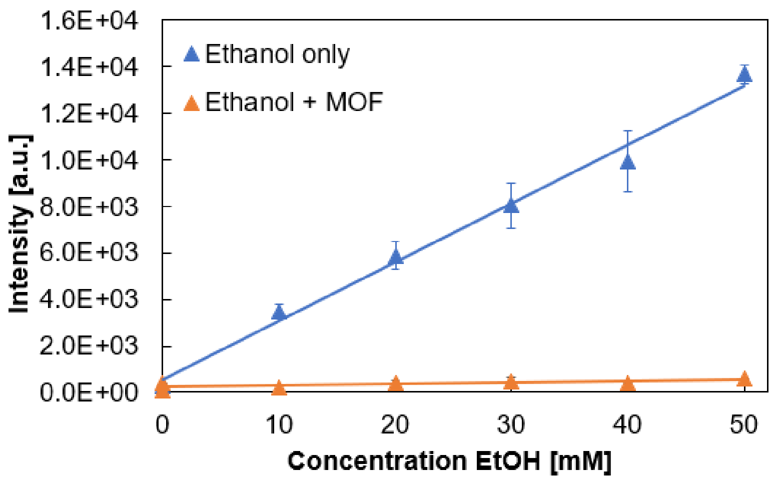

Figure S16. Intensity of *m/z* 47 vs concentration for dilution series of ethanol in water with and without added MOF powder.

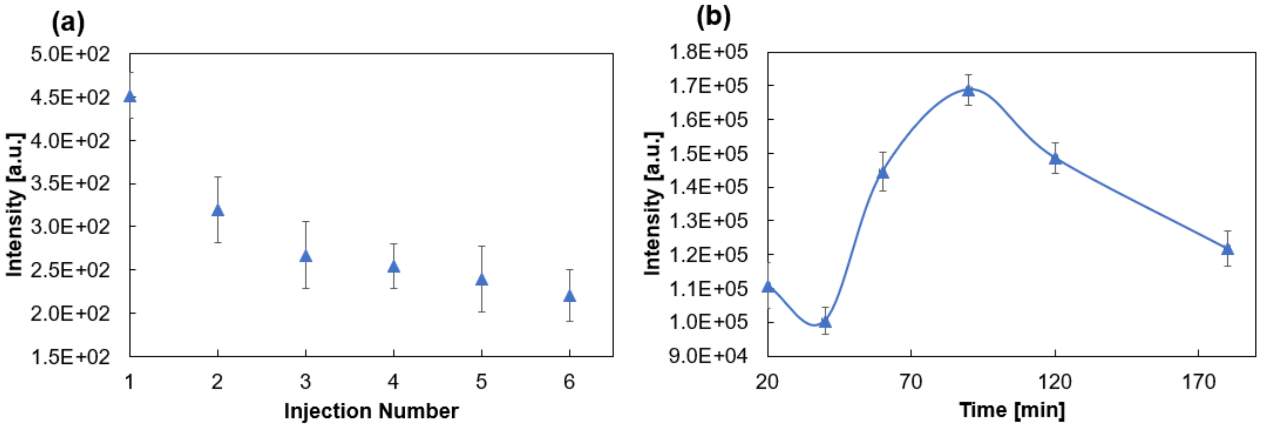

Figure S17. (a) Ethanol peak intensity following repeated sampling of 1 mL headspace from the same closed vial of EtOH solution (1 mM). (b) Measured ethanol intensity vs time for photocatalysed MOF reaction (16 mg/mL MOF, 2 mL total reaction solution volume) with repeat sampling from the same reaction vessel.

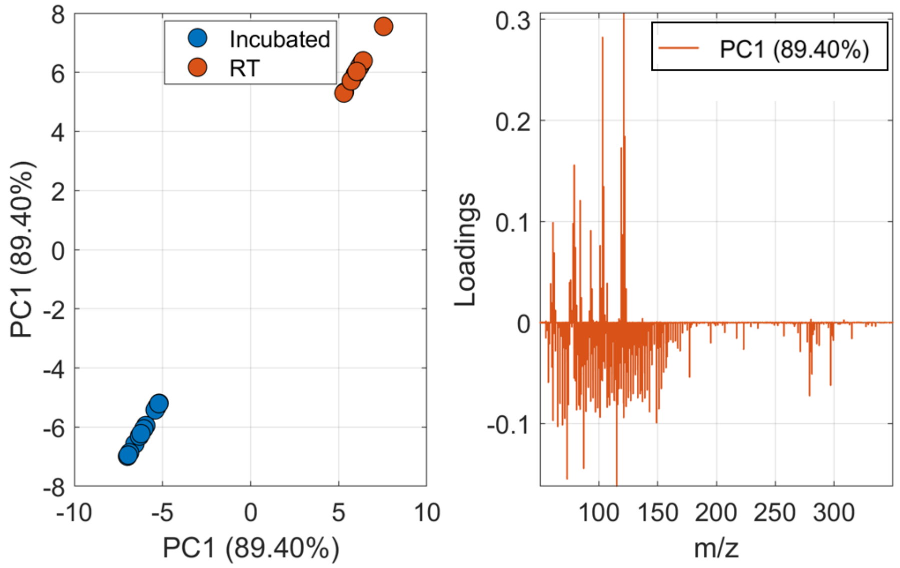

Figure S18. PCA and loadings plot for incubated vs room temperature asparagus urine samples under N_2 atmosphere.
